# *sigE* facilitates the adaptation of *Bordetella bronchiseptica* to stress conditions and lethal infection in immunocompromised mice

**DOI:** 10.1186/1471-2180-12-179

**Published:** 2012-08-16

**Authors:** Sarah E Barchinger, Xuqing Zhang, Sara E Hester, Maria E Rodriguez, Eric T Harvill, Sarah E Ades

**Affiliations:** 1 Department of Biochemistry and Molecular Biology, Pennsylvania State University, 406 Althouse Laboratory, University Park, PA, 16802, USA; 2 Department of Veterinary and Biomedical Sciences, Pennsylvania State University, W210 Millennium Science Complex, University Park, PA, 16802, USA; 3 current address: Department of Microbiology and Immunology, Harvard Medical School, 200 Longwood Ave, Boston, MA, 02115, USA; 4 CINDEFI (UNLP, CONICET La Plata), School of Science, La Plata University, La Plata, Argentina

**Keywords:** *B. bronchiseptica*, Extracytoplasmic function sigma factor, Cell envelope stress, Pathogenesis

## Abstract

**Background:**

The cell envelope of a bacterial pathogen can be damaged by harsh conditions in the environment outside a host and by immune factors during infection. Cell envelope stress responses preserve the integrity of this essential compartment and are often required for virulence. *Bordetella* species are important respiratory pathogens that possess a large number of putative transcription factors. However, no cell envelope stress responses have been described in these species. Among the putative *Bordetella* transcription factors are a number of genes belonging to the extracytoplasmic function (ECF) group of alternative sigma factors, some of which are known to mediate cell envelope stress responses in other bacteria. Here we investigate the role of one such gene, *sigE,* in stress survival and pathogenesis of *Bordetella bronchiseptica*.

**Results:**

We demonstrate that *sigE* encodes a functional sigma factor that mediates a cell envelope stress response. Mutants of *B. bronchiseptica* strain RB50 lacking *sigE* are more sensitive to high temperature, ethanol, and perturbation of the envelope by SDS-EDTA and certain β-lactam antibiotics. Using a series of immunocompromised mice deficient in different components of the innate and adaptive immune responses, we show that SigE plays an important role in evading the innate immune response during lethal infections of mice lacking B cells and T cells. SigE is not required, however, for colonization of the respiratory tract of immunocompetent mice. The *sigE* mutant is more efficiently phagocytosed and killed by peripheral blood polymorphonuclear leukocytes (PMNs) than RB50, and exhibits decreased cytotoxicity toward macrophages. These altered interactions with phagocytes could contribute to the defects observed during lethal infection.

**Conclusions:**

Much of the work on transcriptional regulation during infection in *B. bronchiseptica* has focused on the BvgAS two-component system. This study reveals that the SigE regulon also mediates a discrete subset of functions associated with virulence. SigE is the first cell envelope stress-sensing system to be described in the bordetellae. In addition to its role during lethal infection of mice deficient in adaptive immunity, our results indicate that SigE is likely to be important for survival in the face of stresses encountered in the environment between hosts.

## Background

The cell envelope of bacterial pathogens is critical for survival both in a host during infection and in the environment outside of the host. As the interface between the bacterium and the outside milieu, the cell envelope acts as a barrier protecting the cell against extracellular hazards. Cell envelope structures are also intimately involved in the formation of contacts with host tissues during infection. To safeguard this important compartment, gram-negative bacteria possess an array of stress responses that sense conditions in the cell envelope and alter gene expression to ensure its integrity [1,2]. In many bacterial pathogens, cell envelope stress responses play a multifaceted role. They provide protection against damage caused by components of the immune system, such as complement and antimicrobial peptides that target the cell envelope [3-5]. They regulate the expression of chaperones required for proper assembly of cell envelope-associated structures, including outer membrane porins, pili, and fimbrae [3,6,7]. In addition, cell envelope stress responses can sense the environment around the bacterium and regulate the expression of virulence factors in response to specific cues, ensuring that these factors are expressed at the proper time and location in the host [2,8]. Despite their importance, no cell envelope stress responses have yet been identified or implicated in pathogenesis in *Bordetella* species.

*Bordetella bronchiseptica* is a respiratory pathogen that is closely related to *Bordetella pertussis* and *Bordetella parapertussis*, the causative agents of whooping cough in humans [9,10]. *B. bronchiseptica* causes a range of diseases in various mammals that can be chronic, difficult to completely eradicate, and of variable virulence [11-13]. It is the etiological agent of atrophic rhinitis in swine, kennel cough in dogs, and snuffles in rabbits [12,13]. Documented human infections, generally traced to an animal source, have been observed in immunocompromised individuals, and can be serious, systemic infections [11,14].

The *B. bronchiseptica*, *B. pertussis* and *B. parapertussis* genomes encode a large number of putative transcription factors relative to their overall genome size [15], suggesting that these pathogens have the capacity to extensively regulate gene expression in response to environmental and physiological changes. Despite this finding, only a few *Bordetella* transcription factors have been studied in any detail [16-20]. Among the predicted transcription factors is an ortholog of the cell envelope stress response sigma factor, σ^E^, of *E. coli*. In bacteria, sigma factors are the subunits of bacterial RNA polymerases required for specific promoter recognition and transcription initiation [21]. Alternative sigma factors, like σ^E^, are activated in response to specific stresses and rapidly reprogram gene expression by replacing the housekeeping sigma factor and directing RNA polymerase to the genes in their regulons [21,22].

σ^E^ belongs to the RpoE-like group of extracytoplasmic function (ECF) sigma factors that have been increasingly implicated as key factors contributing to both bacterial stress responses and virulence [23,24]. These sigma factors are widely distributed across bacterial phyla. Where studied, they direct a diverse set of stress responses primarily targeted to the cell envelope [2,8,24,25]. In *E. coli* and *Salmonella enterica* serovar Typhimurium, σ^E^ controls many genes whose products are required for the proper expression of outer membrane porins and LPS [26,27]. During infection, σ^E^ of *S.* Typhimurium is required for survival and proliferation in epithelial and macrophage cell lines, and in the presence of antimicrobial peptides [6,28,29]. In *Pseudomonas aeruginosa*, the σ^E^ homologue, AlgU, controls the expression of the exopolysaccharide alginate and conversion to mucoidy. AlgU is constitutively activated in many clinical isolates from cystic fibrosis patients [30,31]. In addition, σ^E^ is required for the viability of some bacterial species, but not others. The gene encoding σ^E^ is essential in *E. coli* and *Yersinia enterocolitica*, but is dispensable in the closely related species *S.* Typhimurium [6,32,33]. These observations suggest that the functions of σ^E^ orthologs have been adapted to combat the challenges each organism faces in its particular environmental niche. By exploring the role of σ^E^ in diverse bacterial species, we can learn which aspects of this widespread regulatory pathway are universally conserved and which have diverged over the course of evolution.

Here we show that the *B. bronchiseptica* σ^E^ ortholog, encoded by the gene *sigE* (BB3752), is an active sigma factor that mediates a cell envelope stress response. This is the first demonstration of an envelope stress-sensing system in *Bordetella* species. Using a murine infection model, we demonstrate that SigE plays an important role during lethal infection in mice lacking adaptive immunity, but not in respiratory tract colonization. This finding has important implications for human disease, given the observation that *B. bronchiseptica* can cause serious systemic infections in immunocompromised humans [11,14]. This study suggests that SigE is a critical factor in this process, in addition to the BvgAS master virulence regulatory system.

## Results

### *sigE* encodes an active sigma factor

**Figure 1:**
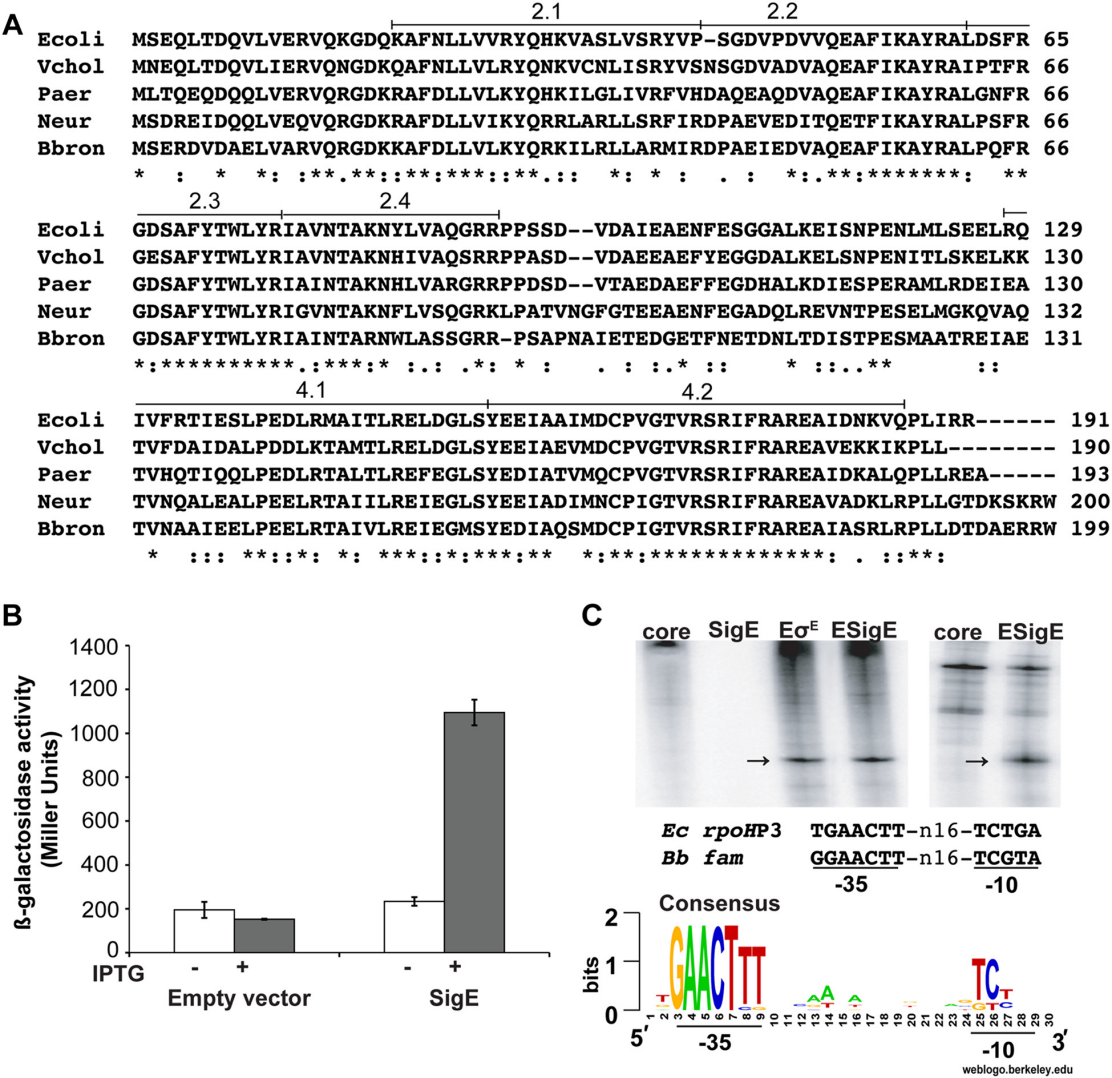
***B. bronchiseptica *SigE is a functional sigma factor.** (**A**) Amino acid sequence alignment of RpoE- like sigma factors from *Escherichia coli* (Ecoli)*, Vibrio cholerae* (Vchol), *Pseudomonas aeruginosa* (Paer), *Nitrosomonas europaea* (Neur) and *B. bronchiseptica* (Bbron) using ClustalW2 (EMBL-EBI). Asterisks indicate identity, two dots indicate strong similarity, and one dot indicates weak similarity between amino acid residues. Conserved sigma factor regions 2.1-2.4 and 4.1-4.2 [22] are indicated above the alignment. Regions 2.3, 2.4, and 4.2 are responsible for promoter recognition [22]. (**B**) β-galactosidase activity from the *E. coli rpoH*P3-*lacZ* reporter increases when *B. bronchiseptica sigE* expression is induced from plasmid pSEB006 in strain SEA5005 by the addition of IPTG. No increase is seen upon IPTG addition to the control strain, SEA008, containing the empty vector. The observed difference in the amount of β-galactosidase activity between the two strains in the presence of IPTG is statistically significant (P value <0.001) (**C**) In vitro transcription from a supercoiled plasmid template containing the *E. coli* σ^E^-dependent *rpoH*P3 promoter with *E. coli* core RNA polymerase (core), SigE alone, Eσ^E^, and ESigE (left panel). In vitro transcription from a linear template containing the promoter region of *B. bronchiseptica fam*, with *E. coli* core RNAP alone (core), or ESigE (right panel). Arrows indicate transcripts from the *rpoH*P3 and *fam* promoters. Below, an alignment of the *E. coli rpoH*P3 and *B. bronchiseptica fam* promoter sequences and a sequence logo showing the consensus promoter for RpoE-like (ECF02) sigma factors from Staron et al. [24].

The *sigE* gene of *B. bronchiseptica* shares a number of conserved residues with other members of the RpoE-like sigma factors, including those in the DNA-binding regions (Figure 1A) [24]. To determine if *sigE* encodes an active sigma factor, we asked whether it could direct transcription from the σ^E^-dependent *rpoH*P3 promoter in *E. coli*. This promoter shares a high degree of similarity with a consensus promoter proposed for the RpoE-like sigma factors that was determined from both experimental data and predicted promoter sequences (Figure 1C) [24,27]. The *sigE* gene from *B. bronchiseptica* strain RB50 was cloned into the pTrc99a expression plasmid and transformed into a derivative of *E. coli* MG1655 that carries an *rpoH*P3::*lacZ* reporter gene fusion integrated on the chromosome [34]. When *sigE* expression was induced, LacZ activity increased, indicating that SigE can initiate transcription from this promoter (Figure 1B). Furthermore, we found that the gene encoding σ^E^, *rpoE*, which is essential for viability in *E. coli*, could be deleted when *sigE* was overexpressed (data not shown, see Materials and Methods).

To provide additional evidence that SigE is a functional sigma factor, N-terminally His-tagged SigE was purified and tested for its ability to initiate transcription in vitro from the *E. coli rpoH*P3 promoter. Holoenzyme formed with SigE and *E. coli* core RNA polymerase (ESigE) was able to direct transcription and produced a transcript of equivalent length to that generated by *E. coli* Eσ^E^ (Figure 1C). The region immediately upstream of the *B. bronchiseptica rpoH* homologue, encoded by the *fam* gene, contains a sequence that is similar to the proposed σ^E^-dependent consensus promoter, suggesting that *B. bronchiseptica rpoH* is regulated by SigE. Indeed, SigE was able to direct transcription from the putative *fam* promoter region in vitro (Figure 1C). Taken together, these results demonstrate that SigE is a functional sigma factor and can initiate transcription from promoter sequences similar to those utilized by other members of the RpoE-like sigma factor family.

### *sigE* contributes to the *B. bronchiseptica* stress response

**Figure 2:**
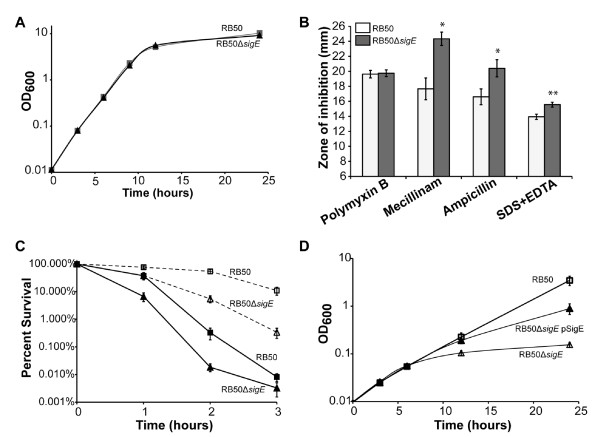
**Role of SigE in response to environmental stresses.** (**A**) RB50 (squares) and RB50Δ*sigE* (triangles) grow similarly at 37°C in Stainer-Scholte broth. (**B**) RB50Δ*sigE* (white bars) is more sensitive than RB50 (grey bars) to treatment with 100 μg mecillinam, 10 μg ampicillin, or 750 μg SDS and 2.9 μg EDTA, but is similarly sensitive to treatment with 300 IU polymyxin B in disk diffusion assays. The average diameters of the zones of inhibition ± SE from at least three independent experiments are shown. The disk diameter was 6 mm. The observed differences between the zones of inhibition for RB50 and the *sigE* mutant are statistically significant for mecillinam, ampicillin, and SDS-EDTA (* indicates a P-value of < 0.05; ** indicates a P-value < 0.01). (**C**) RB50Δ*sigE* (triangles) is more sensitive than RB50 (squares) to heat shock (solid line, filled symbols) caused by shifting cultures from 37°C to 50°C. RB50Δ*sigE* also exhibits reduced thermotolerance (dashed line, open symbols), surviving less well than RB50 when adapted first to 40°C before a shift to 50°C. The mean percent survival±SE of fifteen independent experiments for each strain is shown. (**D**) RB50Δ*sigE* containing the empty cloning vector pEV (open triangles) is more sensitive to treatment with 3% ethanol than RB50 pEV (squares). Expression of plasmid-encoded SigE (RB50Δ*sigE* pSigE) restores growth in 3% ethanol (filled triangles) to near wild-type levels at the 6 and 12 hour time points and partially restores growth at the 24 hour time point. The mean OD_600_ ± SE of at least four independent experiments is shown for each strain.

To investigate the role of SigE in *B. bronchiseptica*, an in-frame deletion of the *sigE* gene was constructed in RB50 (RB50Δ*sigE*) that removed 176 out of 200 codons of the gene, leaving 22 and 2 codons at the 5´ and 3´ ends of the gene, respectively. The deletion was confirmed by PCR and Southern blotting methods (data not shown). σ^E^ orthologs are essential in some bacteria, including *E. coli* and *Y. enterocolitica*[33,35], yet are not required for viability in many other species, such as *S.* Typhimurium, *P. aeruginosa*, and *Burkholderia pseudomallei*[6,36,37]. Deletions of *B. bronchiseptica sigE* were readily obtained, suggesting that it falls in the latter class, and is not essential for viability. Furthermore, RB50Δ*sigE* grew at a rate similar to that of RB50 under standard growth conditions (37°C in Stainer-Scholte broth) (Figure 2A).

To investigate whether SigE mediates a cell envelope stress response in *B. bronchiseptica*, we used disk diffusion assays to compare the sensitivity of RB50 and RB50Δ*sigE* to several chemicals that compromise cell envelope integrity and a series of antibiotics that block different steps in peptidoglycan synthesis. The *sigE* mutant was more sensitive than the wild-type strain to the detergent SDS in combination with EDTA (Figure 2B). The *sigE* mutant was also more sensitive than wild-type RB50 to the antibiotics mecillinam and ampicillin (Figure 2B), whereas sensitivity to meropenem, aztreonam, and imipenem was not affected (data not shown). Unlike σ^E^ orthologs in other bacteria, SigE was not required for resistance to the cationic antimicrobial peptide polymyxin B, which targets bacterial membranes, or to osmotic stress (Figure 2B and data not shown) [6,36,38,39]. RB50Δ*sigE* and RB50 were also equally sensitive to antibiotics that inhibit cytoplasmic processes such as translation (chloramphenicol, erythromycin, kanamycin, tetracycline), transcription (rifampicin), and cytoplasmic enzymes such as DNA gyrase (nalidixic acid), and dihydrofolate reductase (trimethoprim) (data not shown). This lack of sensitivity to multiple antibiotics suggests that the *sigE* mutation does not lead to an overall increase in the permeability of the outer membrane, which would allow more of the antibiotic to enter the cell. These results show that SigE is important for survival in response to specific types of damage to the cell envelope, such as disruption of cellular membranes caused by SDS/EDTA and interference with synthesis of the peptidoglycan layer caused by ampicillin and mecillinam.

We next asked if *sigE* is important for survival following a shift to high temperature, which perturbs both the cell envelope and cytoplasm. RB50 and RB50Δ*sigE* were grown at 37°C to an OD_600_ of 0.4, then shifted to 50°C, a lethal temperature for *B. bronchiseptica*. Cell viability, assessed by CFU/ml, was measured after the shift to 50°C. Survival of the RB50Δ*sigE* strain was lower than that of RB50 (Figure 2C). In attempting to complement this phenotype, we found that plasmid-encoded *sigE* did not restore survival during heat shock (data not shown), although it did complement other phenotypes, as described below. Similar variability in complementation of a σ^E^ mutant by a plasmid-encoded *rpoE* gene has been seen in other bacteria [29,36,40,41]. Work from *Burkholderia cenocepacia* showed that expressing σ^E^ from a plasmid actually increased sensitivity to heat stress [36]. In *S.* Typhimurium, an *rpoE* mutant was sensitive to paraquat and did not survive in stationary phase under anaerobic conditions. Expression of *rpoE* from a plasmid partially complemented the former phenotype, but not the latter [29]. Because the anti-sigma factor that regulates σ^E^ activity was not included in any of these instances, it is likely that proper regulation of SigE activity is required for optimal response to particular stresses, not merely excess SigE activity, complicating complementation experiments.

Another aspect of the classical heat shock response is thermotolerance. When bacteria are exposed to an elevated but nonlethal temperature, heat shock responses are induced, resulting in increased production of chaperones and proteases that refold or degrade unfolded proteins [42]. Consequently, the cells are preloaded with protective factors and exhibit increased survival following a subsequent shift to a lethal temperature [42]. To investigate the role of SigE in this phenomenon, RB50 and RB50Δ*sigE* were grown to an OD_600_ of 0.1 at 37°C, shifted to 40°C for 90 min, then shifted to 50°C. RB50 cultures incubated at 40°C before 50°C survived better at all time points than those directly shifted from 37°C to 50°C. For example, 54% of the RB50 cells pre-adapted at 40°C survived two hours after the shift to 50°C (Figure 2C) compared to 0.1% survival for those shifted directly from 37°C to 50°C (Figure 2C). RB50Δ*sigE* pre-adapted at 40°C also survived better at 50°C than when directly shifted from 37°C to 50°C. However, only 38% of the RB50Δ*sigE* cells survived after one hour (compared to 76% of the wild-type RB50), and 5% survived after two hours at 50°C (Figure 2C). These results demonstrate that *B. bronchiseptica* exhibits a classical thermotolerance response and that SigE contributes to this response.

Both ethanol and heat shock lead to protein unfolding and membrane perturbation and often elicit similar stress responses [43]. To test the role of *sigE* in response to ethanol stress, RB50 and RB50Δ*sigE* were subcultured from mid-exponential-phase cultures into fresh Stainer-Scholte broth with or without 3% ethanol. Both strains grew similarly in medium without ethanol, as noted above. RB50 grew significantly slower in medium containing 3% ethanol than in medium without ethanol (compare the growth curve for RB50 in Figure 2D with that in Figure 2A), but eventually reached a cell density only slightly below that of cultures grown without ethanol. In contrast, the cell density of RB50Δ*sigE* grown in the presence of 3% ethanol never surpassed an OD_600_ of around 0.1, even after 24 hours. Expression of plasmid-encoded *sigE* in RB50Δ*sigE* complemented this phenotype, restoring growth in medium with 3% ethanol to nearly that of RB50 (Figure 2D), indicating that *sigE* is required for survival during ethanol stress.

**Figure 3:**
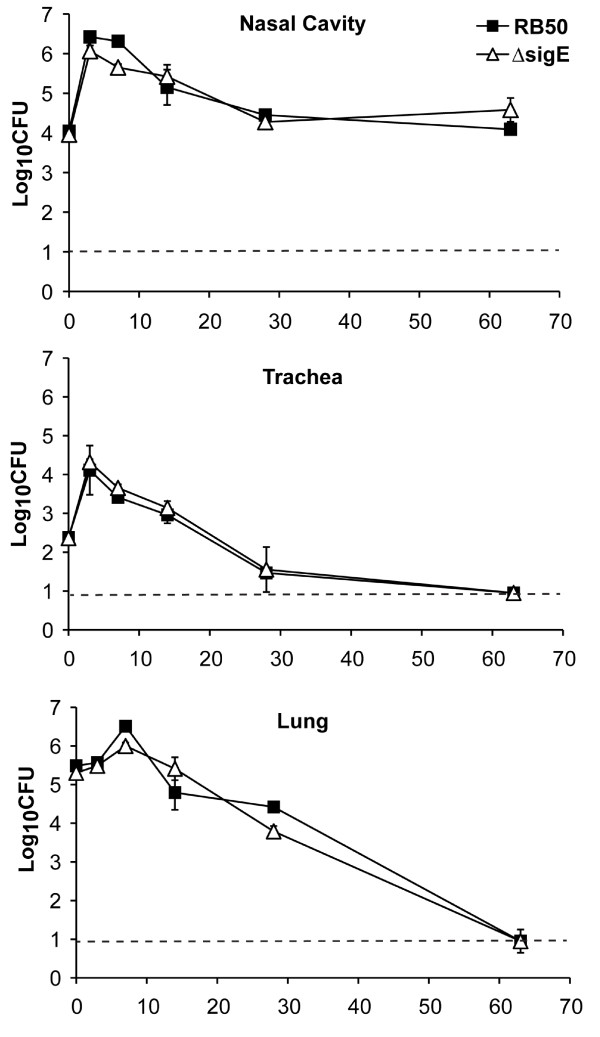
**Colonization of the respiratory tract of C57BL/6 mice by RB50 and RB50Δ*sigE.*** Groups of three 4–6 week-old C57BL/6 mice were inoculated with 5 × 10^5^ CFU of RB50 (filled squares) and RB50Δ*sigE* (open triangles). Groups of three mice were sacrificed at each time point. The bacterial load in the indicated organ is expressed as log_10_ CFU ± SE. The dashed line indicates the limit of detection. The experiment was performed twice with similar results and a representative dataset is shown.

σ^E^ homologues have also been found to play a role during oxidative stress in *S.* Typhimurium and *Burkholderia pseudomallei*[29,41]. However, in disk diffusion assays, SigE was not required for survival in the presence of hydrogen peroxide or paraquat, two inducers of oxidative stress (data not shown). Either SigE is not involved in combating oxidative stress in *B. bronchiseptica,* or other oxidative-stress responsive pathways compensate for SigE when it is absent.

### Growth in the murine respiratory tract is not affected by the lack of *sigE*

*B. bronchiseptica* RB50 colonizes the respiratory tract of immunocompetent mice, causing an asymptomatic infection that is eventually cleared by the immune system. To determine whether *B. bronchiseptica* SigE contributes to colonization and persistence in the respiratory tract, groups of C57BL/6 mice were inoculated with RB50 or RB50Δ*sigE*. Colonization was measured in the nasal cavity, trachea, and lung on days 0, 3, 7, 14, 28 and 63 post-inoculation. Both wild-type and *sigE*-deficient RB50 colonized the nasal cavity at comparable levels, peaking on day 3 post-inoculation, and stabilizing at about 10^4-5^ CFU by 2 weeks post-inoculation (Figure 3). Both strains also showed similar colonization kinetics in the lower respiratory tract of C57BL/6 mice, peaking in numbers on days 3 and 7 post-inoculation in the trachea and lungs, respectively, and declining thereafter, with complete clearance in both organs by day 63 post-inoculation (Figure 3). These data indicate that *B. bronchiseptica* SigE is not required for colonization or persistence in the murine respiratory tract.

### SigE contributes to lethal *B. bronchiseptica* infection in mice lacking B cells and T cells, but not in mice lacking TLR4 or TNF-α

*B. bronchiseptica* has been observed to cause a range of disease including bronchitis, lethal pneumonia, and even systemic infection [11,12]. Mice with defined immune deficiencies are particularly susceptible to different forms of disease [44-46], facilitating assessment of the roles of specific bacterial factors/functions in interactions with different aspects of the host immune response.

Mice lacking key components of innate immunity, either TLR4 or TNF-α, were challenged with RB50 or RB50Δ*sigE* and signs of severe disease were monitored. Consistent with published studies, TLR4^def^ and TNF-α^−/−^ mice inoculated with 10^5^ CFU of RB50 quickly developed signs of lethal bordetellosis such as ruffled fur, hunched posture, decreased activity, and difficulty breathing, and succumbed 2 to 5 days post-inoculation [46,47]. Mice challenged with RB50Δ*sigE* also showed similar signs of disease and time to death (data not shown). In a separate experiment, TLR4^def^ mice and TNF-α^−/−^ mice infected with RB50 or RB50Δ*sigE* that were still alive by day 3 post-inoculation were dissected for bacterial enumeration in the respiratory as well as systemic organs. Both wild-type and *sigE*-deficient RB50 colonized the lungs of TLR4^def^ mice at 10^7-8^ CFU, which was almost 1000-fold higher than in the lungs of TLR4^suf^ mice. Moreover, both strains colonized the systemic organs in TLR4^def^, but not TLR4^suf^ mice (data not shown). Both strains also grew to higher numbers in the lungs of TNF-α^−/−^ mice than in the lungs of C57BL/6 mice and were recovered from systemic organs only in TNF-α^−/−^ mice (data not shown). These data indicate that SigE is not required for *B. bronchiseptica* to cause lethal infection and colonize systemic organs in mice lacking TLR4 or TNF-α.

**Figure 4:**
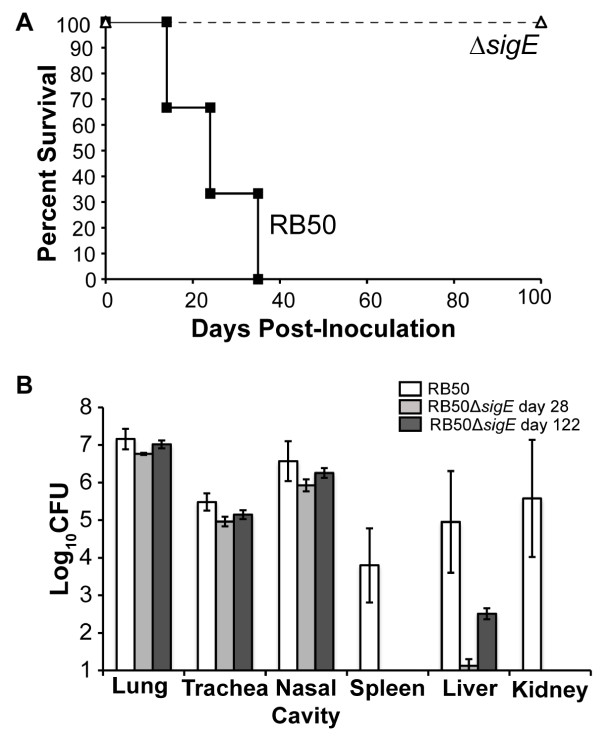
**Survival and systemic colonization of Rag1**^**−/−**^**mice following infection with RB50 and RB50Δ*sigE.*** (**A**) Groups of Rag1^−/−^ mice (n = 6) were inoculated with 5 × 10^5^ CFU of RB50 (solid line with filled squares) or RB50Δ*sigE* (dashed line with open triangles) and monitored for survival. (**B**) Groups of four Rag1^−/−^ mice were inoculated with 5 × 10^5^ CFU of RB50 (white bars) or RB50Δ*sigE* (light grey bars) and dissected on day 28 post-inoculation for bacterial enumeration in the indicated organs. In a separate experiment, Rag1^−/−^ mice inoculated with RB50Δ*sigE* were euthanized for bacterial numbers in the indicated organs on day 122 post-inoculation (dark grey bars). The bacterial load is expressed as log_10_ CFU ± SE. Limit of detection is indicated as the bottom of the y-axis.

B and T cell-deficient Rag1^−/−^ mice succumb to *B. bronchiseptica* infection, and death is associated with systemic spread of the infection [48]. To assess the role of SigE during infection in hosts deficient in adaptive immunity, groups of Rag1^−/−^ mice were inoculated with 5 × 10^5^ CFU of RB50 or RB50Δ*sigE*. Rag1^−/−^ mice inoculated with RB50 showed symptoms of lethal bordetellosis on day 13 post-inoculation and succumbed between days 14–35 post-inoculation (Figure 4A). However, Rag1^−/−^ mice inoculated with RB50Δ*sigE* survived without any overt signs of disease and were euthanized on day 122 post-inoculation. The nasal cavity, trachea, lungs, spleen, liver, and kidneys of these mice were excised to enumerate bacterial loads. Although 10^5-7^ CFU of RB50Δ*sigE* were recovered from the respiratory tract, this strain failed to colonize the spleen or kidney, and only 300 CFU were recovered from the liver (Figure 4B, dark gray bars). In a separate experiment, RB50 and RB50Δ*sigE*-inoculated Rag1^−/−^ mice were sacrificed on day 28 post-inoculation, when some of the RB50-challenged mice were still alive. The bacterial loads of RB50 and RB50Δ*sigE* in the respiratory tract on day 28 post-inoculation were similar, about 10^5-7^ CFU. At this time, 10^4-6^ CFU of RB50 were recovered from liver, spleen, and kidney (Figure 4B, white bars). RB50Δ*sigE*, however, failed to colonize the spleen, kidney or liver (Figure 4B, light gray bars). These results demonstrate that SigE is required for lethal infection by *B. bronchiseptica* in Rag1^−/−^ mice.

The failure of RB50Δ*sigE* to colonize distal organs of Rag1^−/−^ mice suggests that this mutant may be defective in getting into or survival in the bloodstream and/or systemic organs. The bloodstream includes many important bactericidal factors of the host immune system, including complement and phagocytes. We first examined whether *B. bronchiseptica* lacking *sigE* is more susceptible to complement-mediated killing. 500 CFU of RB50, RB50Δ*sigE*, or RB50Δ*wbm*, a strain lacking O-antigen, which is known to be susceptible to complement [48], were incubated at 37°C for one hour in PBS with 20% complement-active or complement-inactive serum from naïve mice. The survival of RB50Δ*sigE* and RB50 was not affected by the presence of either serum (data not shown). In contrast, the RB50Δ*wbm* strain was almost completely killed by complement-active, but not complement-inactive serum (0.7% survival in the presence of complement-active serum compared to 100% survival in the presence of complement-inactive serum). The observation that RB50Δ*sigE* survived in the presence of serum without *B. bronchiseptica*-specific antibodies indicates that the defect in causing systemic infection in mice lacking B and T cells is not due to failure to survive the antimicrobial components in serum, including complement.

### SigE contributes to cytotoxicity to macrophages

**Figure 5:**
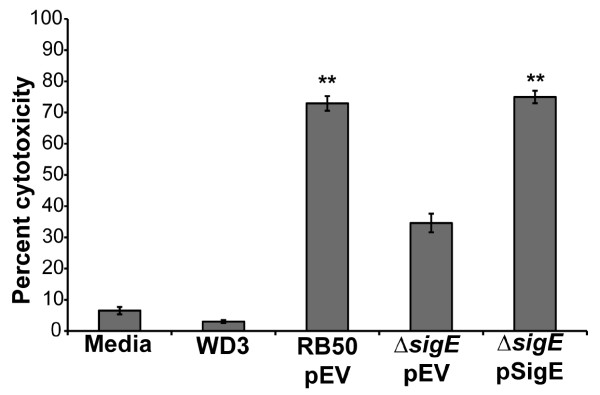
**RB50Δ*sigE *is less cytotoxic to macrophages than RB50.** RAW 264.7 cells were incubated at an MOI of 10 with medium containing RB50 pEV, RB50Δ*sigE* pEV, RB50Δ*sigE* pSigE, TTSS-deficient RB50 strain WD3, or medium alone for 4 hours in the presence of 1 mM IPTG to induce expression of *sigE* from the pLac promoter of pSigE. The average percent cytotoxicity of four wells in four separate experiments as measured by (LDH release from a well/LDH release from the positive control well) x100 ± SE is shown. The differences in percent cytotoxicity between RB50Δ*sigE* pEV and either RB50 pEV or RB50Δ*sigE* pSigE are statistically significant (** indicates P value < 0.01), while the cytotoxicities of RB50 pEV and RB50Δ*sigE* pSigE are not significantly different.

We further tested whether RB50Δ*sigE* interacts differently than RB50 with another major bactericidal component in the bloodstream, phagocytes. *B. bronchiseptica* is cytotoxic to macrophages, and this toxicity has been attributed to the activities of the type three secretion system (TTSS) [49]. To test the role of SigE in macrophage cytotoxicity, RAW264.7 murine macrophages were incubated for 4 hours at an MOI of 10 with RB50, RB50 lacking *sigE*, or RB50 lacking a functional TTSS (WD3). In this experiment, both the RB50 and RB50Δ*sigE* strains contained the empty cloning vector pEV to allow direct comparisons with the complemented strain, RB50Δ*sigE* pSigE. Cytotoxicity was determined by measuring LDH release from the treated macrophages. WD3 caused little cytotoxicity, similar to treatment with medium alone. RB50Δ*sigE* pEV caused approximately 50% less cytotoxicity than wild-type RB50 pEV (Figure 5). This defect in cytotoxicity was complemented by supplying the *sigE* gene on the plasmid pSigE (Figure 5), indicating that loss of *sigE* negatively impacts the ability of RB50 to kill macrophages.

### RB50Δ*sigE* is more efficiently phagocytosed and killed by PMNs

**Figure 6:**
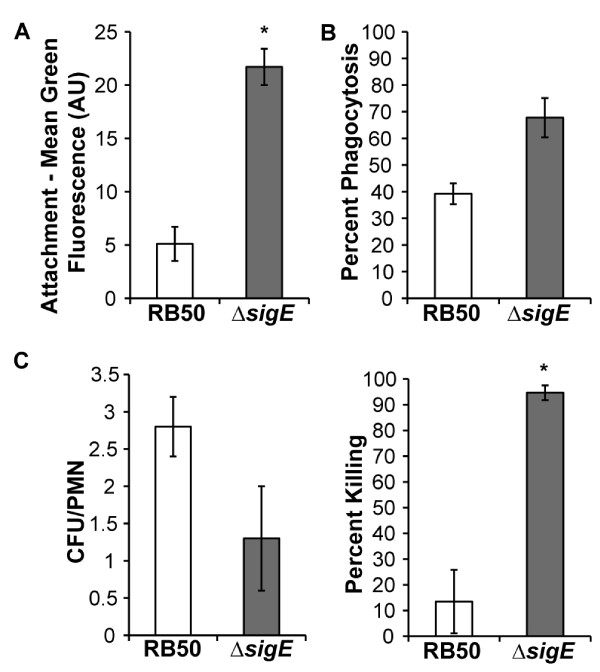
**RB50Δ*sigE *is more efficiently phagocytosed and killed by PMNs than RB50.** (**A**) GFP-expressing RB50 (white bars) and RB50Δ*sigE* (grey bars) were incubated with freshly isolated human peripheral blood PMNs for 20 min at an MOI of 50. Attachment levels were measured as mean intensities ± SE of green fluorescence associated with PMNs. (**B**) Cell surface-bound bacteria were detected by incubation with RPE-labeled goat F(ab’)2 fragments of anti-mouse IgG, after incubation with immune serum. Mean phagocytosis levels ± SE were calculated from the decrease in red fluorescence of GFP-positive cells incubated for an additional 30 min at 37°C allowing for internalization (RPE2, 50 min total incubation time) compared to that of cells incubated for only 20 min (RPE1). Percent phagocytosis is (1-RPE2/RPE1) × 100%. (**C**) To determine killing of bacteria by PMNs, cells incubated with bacteria for 50 min were treated with antibiotics to kill extracellular bacteria. Viable bacteria per PMN (left) and percent killing of internalized bacteria (right) were expressed as mean ± SE. AU indicates arbitrary units; * indicates a P-value of < 0.05.

To test if RB50Δ*sigE* is more susceptible to another bactericidal mechanism, phagocytosis by peripheral blood polymorphonuclear leukocytes (PMNs), RB50 and RB50Δ*sigE* were incubated with freshly isolated human PMNs and attachment to, phagocytosis by, and killing by these cells were measured. PMNs bound RB50Δ*sigE* more efficiently than RB50 (Figure 6A), and significantly more RB50Δ*sigE* than RB50 were phagocytosed by PMNs (Figure 6B). However, the number of viable intracellular RB50Δ*sigE* was ~50% of the numbers of viable RB50 (Figure 6C, left panel). When differences in attachment and phagocytosis were taken into consideration, significantly more internalized RB50Δ*sigE* were killed compared to RB50 (Figure 6C, right panel). Together, these data indicate that SigE contributes to *B. bronchiseptica* resistance to phagocytosis and killing by PMNs.

## Discussion

The BvgAS system of the bordetellae plays a central role in regulating gene expression during pathogenesis [50-52]. However, other regulators may be required during the infectious disease cycle, as *Bordetella* genomes have a large number of putative sensory systems [10,16-20]. In this study, we focused on cell envelope sensing systems and investigated the alternative sigma factor, SigE. We found that SigE of *B. bronchiseptica* does indeed mediate a protective cell envelope stress response and that strains lacking SigE do not establish lethal infections in mice lacking adaptive immunity. These data suggest that the role of SigE is to combat stresses to the envelope imposed by the immune system within a host and by harsh conditions in the environment outside a host. This work is the first demonstration of a cell envelope sensing system in the bordetellae. The σ^E^ system has been explored in the most depth in enteric pathogens belonging to the Gammaproteobacteria [23,25,53]. The bordetellae, members of the Betaproteobacteria, encounter distinctly different environments in the respiratory tract and therefore provide an excellent model to study how the SigE system has been adapted throughout evolution to serve the needs of diverse bacterial pathogens.

The entire *sigE* locus (BB3752-BB3750) is identical at the amino acid sequence level among the classical bordetellae, suggesting a conserved role in the human pathogens *B. pertussis* and *B. parapertussis*. However, the lifestyles and, therefore, conditions encountered differ amongst these three species. *B. bronchiseptica* can live outside the host and primarily infects mammals, although it can infect immunocompromised humans [11,14]. In contrast, *B. pertussis* and *B. parapertussis* primarily infect humans and are directly transmitted between hosts [54,55]. As we learn more about the role of SigE in the bordetellae, it will be of interest to determine whether stresses that induce the SigE system and the SigE regulon members are as highly conserved as the *sigE* locus itself among the bordetellae.

Our results define roles for SigE in *B. bronchiseptica* that are only partially overlapping with those for σ^E^ in other pathogens. SigE was important for survival of *B. bronchiseptica* in the face of both global stresses to the cell envelope caused by heat shock, exposure to ethanol and detergent, and specific stresses caused by several beta-lactam antibiotics (Figure 2). Heat shock, ethanol, and detergent are classical stressors used in the laboratory to mimic conditions that lead to unfolded proteins and disrupted lipids during infection and in the environment. In contrast to the *B. cenocepacia* and *S.* Typhimurium proteins, *B. bronchiseptica* SigE was not required for survival during osmotic stress [6,36]. SigE was also not required for response to oxidative stress or the antimicrobial peptide polymyxin B, unlike the *S.* Typhimurium σ^E^ ortholog [6,29]. The variations among bacteria in their use of σ^E^ systems likely reflect both differences in stresses encountered in environmental reservoirs and in particular host tissues during infection, as well as differences in the arrays of additional cellular stress responses possessed by each species. These other responses can act along with or in place of σ^E^. The presence of other stress responses may be particularly pertinent to *B. bronchiseptica*. Its genome is predicted to encode six related ECF sigma factors of unknown function in addition to SigE [24] that may have complimentary and redundant functions with SigE. Future studies defining conditions that activate other ECF sigma factors and their roles in *B. bronchiseptica* pathogenesis will provide a more comprehensive understanding of how *B. bronchiseptica* copes with extracytoplasmic stress.

Stress response systems, like the σ^E^ system, rapidly induce the expression of specialized sets of genes. These systems are often tightly regulated and expressed only when needed, because inappropriate expression of their regulons can interfere with other important cellular functions [8,56,57]. We found that SigE was not required for colonization and persistence of RB50 within the respiratory tract of an immunocompetent host (Figure 3), the primary niche of *B. bronchiseptica*. This result suggests that the pathogen does not encounter stresses in the respiratory tract that require a response by the SigE system. However, *B. bronchiseptica* encounters different challenges during infection in Rag1^−/−^ mice lacking B and T cells. In these mice, the infection spreads to the bloodstream, which is under greater immune surveillance and has a different arsenal of antimicrobial factors to attack invaders than the respiratory tract. The defect of RB50Δ*sigE* in lethal infection of Rag1^−/−^ mice, therefore, reveals a specific function for SigE in response to an unknown stress, possibly related to the innate immune response, that the bacteria encounter during infections that proceed beyond colonization of the respiratory tract.

The inability of RB50Δ*sigE* to cause lethal infections in Rag1^−/−^ mice (Figure 4) could be due to failure to enter or survive in the bloodstream and/or systemic organs of these mice. Since the mutation does not affect survival during incubation with serum in vitro, it is unlikely that the *sigE*-deficient strain is more susceptible to complement or other antimicrobial components in serum. The defect in infection of Rag1^−/−^ mice may then be related to altered interactions of the mutant strain with phagocytic cells in the bloodstream. RB50Δ*sigE* is more susceptible to peripheral blood PMNs than RB50 (Figure 6), and is also less cytotoxic to macrophages than RB50 (Figure 5). Either or both of these defects could explain the failure to recover RB50Δ*sigE* from systemic organs of mice lacking adaptive immune responses and the decreased virulence in these mice.

Why does the RB50Δ*sigE* mutant spread systemically and cause lethal infection in TLR4^def^ and TNF-α^−/−^ mice, but not Rag1^−/−^ mice? The lower cytotoxicity of the *sigE* mutant and its increased sensitivity to phagocytic killing does not affect its virulence in mice lacking innate immune functions. This could be because bacterial numbers within the respiratory tract of TLR4^def^ or TNF-α^−/−^ mice are nearly an order of magnitude higher than in the lungs of Rag1^−/−^ mice. As such, the large number of bacteria in TLR4^def^ or TNF-α^−/−^ mice may overwhelm limiting host antimicrobial defense mechanisms that can contain the lower bacterial numbers in the lungs of Rag1^−/−^ mice. Alternatively, although the cytotoxicity of the *sigE* mutant is reduced, it may still be sufficient to establish lethal infections in the absence of TLR4 or TNF-α. Thus TLR4- and TNF-α-dependent functions, such as efficient phagocytosis and killing, appear to be sufficient to prevent lethal infection by RB50Δ*sigE* in Rag1^−/−^ mice. Although the exact role remains to be elucidated, our results clearly indicate that SigE is required for lethal infection of mice lacking B and T cells.

Although the *B. bronchiseptica* strain RB50 causes asymptomatic infections in immunocompetent mice, other strains of *B. bronchiseptica* can cause a wide range of disease severity in other hosts [11-13]. In particular subsets of immunocompromised humans, such as those infected with HIV, severe systemic *B. bronchiseptica* infections have been observed [14]. These facts, along with the high degree of sequence conservation for the *sigE* locus in *B. pertussis* and *B. parapertussis*, highlights the importance of understanding the stressors that activate SigE and how the SigE system responds to them during infection.

## Conclusions

In this work, we have demonstrated that the *B. bronchiseptica* extracytoplasmic function sigma factor, SigE, is important for surviving global stresses that affect the whole cell, such as heat shock and ethanol stress, specific stresses that target the cell envelope, such as beta-lactam antibiotics and SDS-EDTA, and in interactions with the host innate immune system, particularly phagocytes. During infection, SigE is not required for colonization of the respiratory tract of immunocompetent mice. However, it is needed for a specific set of functions associated with virulence, particularly those involved in surviving the innate immune response when the infection progresses in immunocompromised mice. Although SigE systems are widely conserved, the details as to which aspects are shared and which have diverged are complex*.* As evidence accumulates from studies in different bacteria, it is becoming apparent that these sensory modules are important for stress survival, particularly with respect to the cell envelope. However, the nature of the stresses that SigE systems combat varies. During infection, comparisons are even more difficult, since differences are seen not only amongst SigE systems from one pathogen to another, but also within different niches in the host or during the progression of disease for a single pathogen.

## Methods

### Strains and media

A complete list of strains used in this study can be found in Table 1. *B. bronchiseptica* strains are derivatives of the previously described *B. bronchiseptica* strain RB50 [58]. *B. bronchiseptica* was maintained on Bordet-Gengou (BG) agar (Difco) containing 10% defibrinated sheep blood (Hema Resources) and 20 μg/ml streptomycin. In liquid culture, *B. bronchiseptica* was grown in Stainer-Scholte broth [59] with aeration. Chloramphenicol was used at 20 μ/ml and IPTG at 1 mM where noted. The RB50Δ*sigE* mutant was constructed as described below. *E. coli* strains used to measure SigE activity are derivatives of MG1655 that carry the σ^E^-dependent *rpoH*P3::*lacZ* reporter (strain SEA001 [34]). *E. coli* strain BL21(DE3) pLysS was used to express constructs for protein purification. *E. coli* were grown in LB broth in a gyratory water bath with aeration. Ampicillin was used at 100 μg/ml, tetracycline at 20 μg/ml, and kanamycin at 15 μg/ml as needed for experiments with *E. coli*.

**Table 1:** Strains and plasmids

	**Strain name**	**Genotype**	**Source, Reference**
*E. coli*	SEA001	MG1655 Φλ*rpoH*P3::*lacZ* Δ*lac*X74	[60]
	SEA5036	BL21(DE3) Δ*slyD*::kan pLysS pPER76	[61]
	XQZ001	BL21(DE3) Δ*slyD*::kan pLysS pXQZ001	This work
	SEA4114	CAG43113 Δ*rpoE*::kan Δ*nadB*::Tn*10*	[62]
	SEA008	SEA001 pTrc99a	[62]
	SEA5005	SEA001 pSEB006	This work
	XQZ003	DH5α pXQZ0003	This work
	SS1827	DH5α pSS1827	[63]
*B. bronchiseptica*	RB50	RB50	[58]
	SEA5516	RB50Δ*sigE*	This work
	MER001	RB50 pCW505	This work
	MER002	RB50Δ*sigE* pCW505	This work
	SEA5518	RB50 pEV	This work
	SEA5520	RB50Δ*sigE* pEV	This work
	SEA5526	RB50 pSigE	This work
	SEA5530	RB50Δ*sigE* pSigE	This work
	RB50Δwbm	RB50Δ*wbmBwbmCwbmDwbmE*	[64]
	WD3	RB50Δ*bscN*	[49]
	**Plasmid name**	**Description**	**Source, Reference**
	pTrc99a	Vector, pBR322 ori, Ap^R^	Pharmacia
	pSEB006	*sigE* in pTrc99a	This work
	pSEB015	isolated *rpoH*P3 promoter in pRLG770, Ap^R^	[61]
	pPER76	*rpoE* in T7 expression vector pET15b, Kan^R^	[65]
	pXQZ001	*sigE* in T7 expression vector pET15b, Kan^R^	This work
	pXQZ002	Δ*sigE* in TOPO-TA vector	This work
	pSS1827	helper plasmid competent for mating, Ap^R^	[63]
	pSS3962	*Bordetella*-specific allelic exchange vector, Kan^R^	Stibitz, unpublished work
	pXQZ003	Δ*sigE* in pSS3962	This work
	pEV	Vector pJS72, ΩSpec^R^ cassette replaced with Cm^R^	This work
	pSigE	*sigE* in pEV	This work
	pCW505	cytoplasmic expression of GFP	[66]

### Plasmid constructions

All plasmids used in this study are listed in Table 1 and oligonucleotide sequences are given in Table 2. Plasmid pSEB006 was constructed to express *sigE* in *E. coli*. The *sigE* gene was amplified from RB50 genomic DNA with the primers SigEF and SigER and cloned into the expression vector pTrc99a under the control of the IPTG-inducible trc promoter. To facilitate purification of SigE, the plasmid pXQZ001 was constructed by amplifying the *sigE* gene from RB50 genomic DNA using the primers HisSigEF and HisSigER. The resulting PCR product was cloned into the T7 expression vector pET-15b (Novagen), which adds a 6X-His tag to the N-terminus of recombinant proteins. To express *sigE* in *B. bronchiseptica*, *sigE* was amplified from RB50 genomic DNA using primers 72SigEF and 72SigER and ligated into the XbaI and XhoI sites downstream of the pLac promoter in pEV to create pSigE. The expression vector pEV was constructed from the broad host range vector pJS72 by replacing the spectinomycin resistance gene with the *cat* gene encoding chloramphenicol resistance amplified from pKD3 [67] using primers 72ChlorF and 72ChlorR. The exchange of drug markers was necessary because RB50 is naturally resistant to spectinomycin. pEV and pSigE were moved into RB50 and RB50Δ*sigE* through tri-parental mating on BG agar with MgCl_2_. Transconjugants were selected on BG containing 60 μg/ml streptomycin and 20 μg/ml chloramphenicol. Plasmid pCW505 (kindly supplied by Dr. Alison Weiss, Cincinnati, Ohio), which induces cytoplasmic expression of GFP without affecting growth or antigen expression, was used to visualize RB50 and RB50Δ*sigE* in the phagocytosis assays described below [68].

**Table 2:** Primer sequences

**Primer name**	**Sequence (5´ - 3´)**	**Source or Reference**
SigEF	GGCGGAGAATTCAGGAGGAGGCGTCATGAGCGAACGCGATG	This work
SigER	GGCCTAGGATCCTTACCAGCGACGCTCGGCAT	This work
HisSigEF	GGCCTGGCATATGAGCGAACGCGATGTCGA	This work
HisSigER	GGCCTAGGATCCTTACCAGCGACGCTCGGCAT	This work
72SigEF	GCGCGGTCTAGAAGGAGGAGGCGTCATGAGCGAACGCGATG	This work
72SigER	GCCCGGCTCGAGTTACCAGCGACGCTCGGCAT	This work
72ChlorF	GCGGCGGGATCCTGTGTAGGCTGGAGCTGCTTC	[67]
72ChlorR	GCCGCCGGATCCCATATGAATATCCTCCTTA	[67]
SigEKO_LeftF	GGGAATTCAAGATCGAGATCGGCCTGTCGAAT	This work
SigEKO_LeftR	AGGGATCCGAAGGCTTTCTTGTCGCCACGTTGTA	This work
SigEKO_RightF	AGGGATCCTGGTAAGGAGTGGCAGTCATGCAA	This work
SigEKO_RightR	GCGAATTCAAAGCAACGGTGTCATCAACGTCC	This work
PFamF	GGGCGGGAATTCTGCCGTTCGTGGATGTCCAG	This work
PFamR	GGGCGGAAGCTTGGGCCAACGAACTACTGGGT	This work

### Construction of RB50Δ*sigE* strain

The *sigE* gene was deleted from RB50 using a *Bordetella*-specific allelic exchange procedure to produce strain SEA5516. Primers used in the construction are listed in Table 2. A PCR product containing 637 bp proximal to the 5' end of *sigE* was amplified from RB50 genomic DNA using primers SigEKO_LeftF and SigEKO_LeftR. A non-overlapping PCR product containing 534 bp proximal to the 3' end of *sigE* was amplified with primers SigEKO_RightF and SigEKO_RightR. The two fragments were digested with BamHI and ligated. The resulting construct was amplified with primers SigEKO_LeftF and SigEKO_RightR, cloned into the TopoTA vector (Invitrogen), and verified by sequencing to give plasmid pXQ002. In this deletion construct, the 528 bp central region of the *sigE* gene is deleted leaving 66 bp at the 5' end and 6 bp at the 3' end of the *sigE* gene. The deletion construct from pXQ002 was then cloned into the EcoRI site of the allelic exchange vector pSS3962 (Stibitz S., unpublished data) to generate pXQ003 and transformed into *E. coli* strain DH5α. Tri-parental mating with wild-type *B. bronchiseptica* strain RB50, *E. coli* strain DH5α harboring the pXQ003 vector (strain XQ003), and DH5α harboring the helper plasmid pSS1827 (strain SS1827) [69,70] and selection of mutants were performed as previously described [69]. The deletion strain was verified by PCR using primers SigEKO_LeftF and SigEKO_RightR and by Southern blot analysis.

### β-galactosidase assays

Overnight cultures were diluted into fresh medium and grown to an OD_600_ of 0.1-0.2 at 30°C. Where indicated, IPTG was added to a final concentration of 1 mM. Samples were collected 2.5 hours later and β-galactosidase activity from the σ^E^-dependent reporter was assayed as previously described [60,71].

### Complementation of *E. coli* Δ*rpoE* by *B. bronchiseptica sigE*

The ability of *B. bronchiseptica sigE* to suppress the lethality caused by deletion of *rpoE* in *E. coli* was determined using a cotransduction assay as described [62]. The Δ*rpoE*::*kan* Δ*nadB*::Tn*10* allele from strain SEA4114 was moved via P1 transdution into strain SEA5005, which carries *sigE* on the plasmid pSEB006. Tet-resistant (tet^R^) transductants were selected and then screened for kanamycin resistance (kan^R^). Although the *nadB* and *rpoE* alleles are tightly linked (>99%), cotransduction resulting in tet^R^ kan^R^ colonies will only occur if *rpoE* is no longer essential for viability. In transductions with *E. coli* expressing *sigE* (strain SEA5005) as the recipient strain, 31 out of 32 tet^R^ transductants were also kan^R^. In contrast, none of the 39 tet^R^ transductants were kan^R^ when *E. coli* carrying the empty cloning vector (strain SEA008) was the recipient strain.

### Protein purification

N-terminally His-tagged *B. bronchiseptica* SigE and *E. coli* σ^E^ were purified from strain XQZ001 and SEA5036, respectively, as previously described for *E. coli* σ^E^[61]. Briefly, cells were grown at 25°C to an OD_600_ of 0.5, at which point IPTG was added to induce protein production. Following 1.5-3 hours of induction, cells were harvested by centrifugation and resuspended in lysis buffer (20 mM Tris–HCl pH 8.0, 500 mM NaCl, 20 mM imidazole, 2.5 mM β-mercaptoethanol, 1 mM PMSF). Resuspended cells were then lysed by sonication, and the lysate cleared by centrifugation. The supernatant containing soluble His-SigE was loaded onto a Ni-NTA column (Qiagen). Bound proteins were eluted with a stepwise gradient of 20, 60, 100, and 200 mM imidazole in column buffer (20 mM Tris–HCl pH 8.0, 500 mM NaCl, 2.5 mM β–mercaptoethanol). Fractions containing SigE were pooled and dialyzed into 20 mM Tris–HCl pH 8.0, 50 mM NaCl, and 2.5 mM β-mercaptoethanol.

### In vitro transcription

100 nM *E. coli* core RNA polymerase (Epicentre) was incubated with 400 nM His-SigE or His-σ^E^ in transcription buffer (40 mM Tris–HCl pH 8.0, 10 mM MgCl_2_, 50 mM NaCl, 1 mM DTT, 0.1 μ/ml BSA) for 10 min at 30°C to form holoenzyme. Multi-round transcription reactions were initiated by addition of holoenzyme at a final concentration of 40 nM sigma factor and 10 nM core RNA polymerase, to prewarmed (30°C) transcription mix containing 5.0 nM supercoiled plasmid template pSEB015 [61] or 5.0 nM linear P*fam* template, 5% glycerol, 200 mM ATP, 200 mM CTP, 200 mM GTP, 10 mM UTP, and 2.5 mCi [α-^32^P]UTP in transcription buffer. After 10 min at 30°C, reactions were stopped by the addition of stop solution (80% formamide, 20 mM EDTA, 0.1% xylene cyanol, and 0.1% bromophenol blue). Samples were electrophoresed on 6% polyacrylamide gels containing 7.5 M urea, and transcripts were visualized by phosphorimaging. The linear P*fam* template was generated by amplification of the promoter region of the gene encoding σ^32^ in RB50, *fam*, using the primers PFamF and PFamR (Table 2). The sequence logo in Figure 1C was generated using WebLogo version 2.8.2 ( http://WebLogo.berkeley.edu, [72]).

### Disk diffusion assays

*B. bronchiseptica* cultures in mid-log phase were diluted to 6 × 10^8^ CFU/ml and spread on Stainer-Scholte agar plates to generate a lawn of bacteria. Disks containing 300 IU polymyxin B, 10 μg ampicillin, 100 μg mecillinam, 750 μg sodium dodecyl sulfate (SDS) and 2.9 μg EDTA, 30 μg aztreonam, 10 μg imipenem, 10 μg meropenem, 30 μg chloramphenicol, 15 μg erythromycin, 30 μg kanamycin, 30 μg nalidixic acid, 150 μg rifampicin, 23.75 μg sulfamethoxazole and 1.25 μg trimethoprim, 30 μg tetracycline, 3.0 μg deoxycholate, 3% hydrogen peroxide, or 2% paraquat were applied to the plates and the zones of inhibition were measured after overnight incubation at 37°C.

### Temperature and ethanol stress

For temperature stress experiments, mid-log phase cultures of RB50 and RB50Δ*sigE* were diluted to an OD_600_ of 0.01 in fresh Stainer-Scholte broth and incubated at 37°C in a gyratory water bath with shaking. At an OD_600_ of 0.1, cultures were either shifted to 40°C for adaptation or kept at 37°C. After 90 minutes, all cultures were shifted to 50°C, and survival was measured by plating and CFU counts. For ethanol stress experiments, mid-log-phase cultures of the pertinent strains were subcultured into fresh Stainer-Scholte broth with or without 3% ethanol and incubated at 37°C in a gyratory water bath with aeration. Bacterial growth was measured by OD_600_.

### Complement killing assay

Complement killing assays were performed as previously described [73]. Approximately 500 CFU of RB50, RB50Δ*sigE*, and RB50Δ*wbm* from mid-log phase cultures were incubated with 45 μl of diluted serum from C57BL/6 mice or PBS (final volume for incubation was 50 μl) for 1 hour at 37°C. Bacterial numbers before and after incubation were determined by plating and CFU counts. Each strain was assayed in triplicate.

### Cytotoxicity assay

Cytotoxicity assays were performed as previously described [44]. Briefly, bacteria were added to RAW 264.7 murine macrophage cells at a multiplicity of infection (MOI) of 10 and incubated for four hours. Percent lactate dehydrogenase (LDH) release, a measure of cytotoxicity, was determined by using Cytotox96 Kit (Promega) according to the manufacturer’s protocol.

### Phagocytosis and killing by polymorphonuclear leukocytes

Attachment and phagocytosis of the *B. bronchiseptica* strains by peripheral blood polymorphonuclear leukocytes (PMNs) were evaluated as previously described with a few modifications [74]. Briefly, GFP-expressing bacteria were incubated with PMNs at an MOI of 50 for 20 min at 37°C to allow binding. After extensive washing to remove non-attached bacteria, an aliquot was maintained on ice to be used as a bacterial attachment control. The remaining PMNs were further incubated for 30 min at 37°C to allow internalization, at which point phagocytosis was stopped by placing PMNs on ice. Bacteria bound to the cell surface in both aliquots were detected by incubation with RB50 immune serum for 30 min at 4°C, followed by incubation with R-phycoerythrin (RPE)–labeled goat F(ab')_2_ fragments of anti-mouse IgG at 4°C for 30 min. All incubations were done in the presence of 25% heat-inactivated human serum to prevent nonspecific binding of antibodies. After washing, ten thousand cells per sample were analyzed by flow cytometry. Attachment control samples were also analyzed by fluorescence microscopy using a DMLB microscope coupled to a DC 100 camera (Leica Microscopy Systems Ltd.). Green fluorescence intensity associated with PMNs maintained at 37°C for 20 min has previously been shown to represent bacterial attachment [74]. Phagocytosis was calculated from the decrease in mean red fluorescence intensity of GFP-positive PMNs after the 30 min incubation allowing for internalization, as previously described [75]. Percent phagocytosis was calculated as follows: 100 × (1-RPE2/RPE1), where RPE1 is the mean RPE-fluorescence of the GFP-positive cells after 20 min at 37°C (attachment control) and RPE2 is the mean RPE-fluorescence of the GFP-positive cells after 50 min (internalized bacteria) at 37°C.

Killing of bacteria by PMNs was assessed as follows: after phagocytosis of the bacteria, 400 μg/ml polymyxin B and 350 μg/ml chloramphenicol were added to the PMNs for 1 hour to kill the remaining extracellular bacteria and assess intracellular survival. Serial dilutions of samples were plated to determine the number of viable intracellular bacteria per PMN. The relative percent survival of internalized bacteria was calculated from the relative phagocytosis index and taking into account the initial attachment level of each strain, as follows: percent bacterial killing = [1-N/(A × P)] × 100, where A = number of bacteria associated with PMN after 20 min at 37°C (determined by fluorescent microscopy), P = phagocytosis index (1-RPE2/RPE1), N = number of viable bacteria per cell after incubation with antibiotics. Control experiments to assess the efficacy of antibiotic bactericidal activity were performed in parallel. Briefly, samples of 5 × 10^8^ bacteria were incubated with antibiotics for 30 min at 37°C and plated. This resulted in a >99% decrease in CFU.

### Animal experiments

C57BL/6J, B6.129 S-*Tnf*^tm1Gkl^/J (TNF-α^−/−^), B6 129S7-*Rag*1^tm1Mom^/J (Rag1^−/−^), C3H/HeOuJ (TLR4^suf^) and C3H/HeJ (TLR4^def^) mice were obtained from Jackson laboratories (Bar Harbor). All mice were bred in our *Bordetella*-free, specific pathogen-free breeding rooms at The Pennsylvania State University. For inoculation, mice were sedated with 5% isoflurane (Abbott laboratory) in oxygen and 50 μl of PBS containing 10^5^ or 5 × 10^5^ CFU of the indicated bacteria were pipeted onto the external nares [76,77]. This method reliably distributes the bacteria throughout the respiratory tract [76]. Survival curves were generated by inoculating TLR4^def^, TNF-α^−/−^ and Rag1^−/−^ mice with either RB50 or RB50Δ*sigE*. Mice suffering from lethal bordetellosis as determined by severe hunched posture, ruffled fur, extremely labored breathing and apathy were euthanized to prevent unnecessary suffering [47]. For quantifying bacterial load, mice were euthanized via CO_2_ inhalation, and lung, trachea, nasal cavity, spleen, liver and/or kidneys were excised. Tissues were homogenized in PBS, aliquots were serially diluted, plated, incubated at 37°C for 2 to 3 days, and CFU were determined. All protocols were reviewed by the university IACUC and all animals were handled in accordance with institutional guidelines (IACUC approval number: 31297).

### Statistical analysis

The mean +/− standard error (SE) of the geometric mean was determined when appropriate and expressed as error bars. Two-tailed, unpaired Student’s T-tests were used to determine statistical significance between groups. All experiments were performed at least twice with similar results.

## Authors’ contributions

SB and SA conceived and designed the molecular and stress experiments, which were performed by SB. XZ and EH conceived and designed the infection studies, which were performed by XZ. SH performed the cytotoxicity experiments and MR performed the phagocytosis experiments. SB, XZ, EH, and SA wrote the manuscript. All authors have read, contributed to editing, and approved the final manuscript.
